# Patient survival after D _1_ and D _2_ resections for gastric cancer: long-term results of the MRC randomized surgical trial

**DOI:** 10.1038/sj.bjc.6690243

**Published:** 1999-03

**Authors:** A Cuschieri, S Weeden, J Fielding, J Bancewicz, J Craven, V Joypaul, M Sydes, P Fayers, for the Surgical Co-operative Group

**Affiliations:** University Department of Surgery, Ninewells Hospital and Medical School, Dundee, DD1 9SY, UK; Cancer Division, MRC Clinical Trials Unit, Cambridge, UK; Queen Elizabeth Hospital, Queen Elizabeth Hospital, Birmingham, UK; University Department of Surgery, Hope Hospital, Salford, UK; Kingstown General Hospital, Kingstown General Hospital, St Vincents, Jamaica

**Keywords:** gastric cancer, D _1_ resection, D _2_ resection, long-term survival

## Abstract

Controversy still exists on the optimal surgical resection for potentially curable gastric cancer. Much better long-term survival has been reported in retrospective/non-randomized studies with D _2_ resections that involve a radical extended regional lymphadenectomy than with the standard D _1_ resections. In this paper we report the long-term survival of patients entered into a randomized study, with follow-up to death or 3 years in 96% of patients and a median follow-up of 6.5 years. In this prospective trial D _1_ resection (removal of regional perigastric nodes) was compared with D _2_ resection (extended lymphadenectomy to include level 1 and 2 regional nodes). Central randomization followed a staging laparotomy.

Out of 737 patients with histologically proven gastric adenocarcinoma registered, 337 patients were ineligible by staging laparotomy because of advanced disease and 400 were randomized. The 5-year survival rates were 35% for D _1_ resection and 33% for D _2_ resection (difference –2%, 95% CI = –12%–8%). There was no difference in the overall 5-year survival between the two arms (HR = 1.10, 95% CI 0.87–1.39, where HR > 1 implies a survival benefit to D _1_ surgery). Survival based on death from gastric cancer as the event was similar in the D _1_ and D _2_ groups (HR = 1.05, 95% CI 0.79–1.39) as was recurrence-free survival (HR = 1.03, 95% CI 0.82–1.29). In a multivariate analysis, clinical stages II and III, old age, male sex and removal of spleen and pancreas were independently associated with poor survival. These findings indicate that the classical Japanese D _2_ resection offers no survival advantage over D _1_ surgery. However, the possibility that D _2_ resection without pancreatico-splenectomy may be better than standard D _1_ resection cannot be dismissed by the results of this trial. © 1999 Cancer Research Campaign


*British Journal of Cancer* (1999) **79**(9/10), 1522–1530
© 1999 Cancer Research Campaign
Article no. bjoc.1998.0243

### Patient survival after $D_1$ and $D_2$ resections for gastric cancer: long-term results of the MRC randomized surgical trial

**A Cuschieri[1], S Weeden[2], J Fielding[3], J Bancewicz[4], J Craven[5], V Joypaul[1], M Sydes[2] and P Fayers[2], for the Surgical Co-operative Group**

[1]University Department of Surgery, Ninewells Hospital and Medical School, Dundee DD1 9SY, UK; [2]Cancer Division MRC Clinical Trials Unit, Cambridge, UK; [3]Queen Elizabeth Hospital, Birmingham, UK; [4]University Department of Surgery, Hope Hospital, Salford, UK; [5]Kingstown General Hospital, St Vincents, Jamaica

**Summary** Controversy still exists on the optimal surgical resection for potentially curable gastric cancer. Much better long-term survival has been reported in retrospective/non-randomized studies with $D_2$ resections that involve a radical extended regional lymphadenectomy than with the standard $D_1$ resections. In this paper we report the long-term survival of patients entered into a randomized study, with follow-up to death or 3 years in 96% of patients and a median follow-up of 6.5 years. In this prospective trial $D_1$ resection (removal of regional perigastric nodes) was compared with $D_2$ resection (extended lymphadenectomy to include level 1 and 2 regional nodes). Central randomization followed a staging laparotomy.

Out of 737 patients with histologically proven gastric adenocarcinoma registered, 337 patients were ineligible by staging laparotomy because of advanced disease and 400 were randomized. The 5-year survival rates were 35% for $D_1$ resection and 33% for $D_2$ resection (difference –2%, 95% CI = –12%–8%). There was no difference in the overall 5-year survival between the two arms (HR = 1.10, 95% CI 0.87–1.39, where HR > 1 implies a survival benefit to $D_1$ surgery). Survival based on death from gastric cancer as the event was similar in the $D_1$ and $D_2$ groups (HR = 1.05, 95% CI 0.79–1.39) as was recurrence-free survival (HR = 1.03, 95% CI 0.82–1.29). In a multivariate analysis, clinical stages II and III, old age, male sex and removal of spleen and pancreas were independently associated with poor survival. These findings indicate that the classical Japanese $D_2$ resection offers no survival advantage over $D_1$ surgery. However, the possibility that $D_2$ resection without pancreatico-splenectomy may be better than standard $D_1$ resection cannot be dismissed by the results of this trial.

**Keywords:** gastric cancer; $D_1$ resection; $D_2$ resection; long-term survival

Carcinoma of the stomach remains a major cause of death in most Western countries. The only proven effective therapy is surgery, but overall 5-year survival rates remain low after resection. In 1981, the Japanese Society for Research in Gastric Cancer (JSRGC) standardized the gastric resections and the extent of regional lymphadenectomy in accordance with specific rules (updated over the years) based on the location of the tumour and the respective regional node drainage (Kajitani, 1981). Large retrospective series from Japan of radical gastrectomy with level-2 extended lymphadenectomy ($D_2$ resections) have shown impressive 5-year survival rates, certainly much higher than experienced in the West (Mine et al, 1970; Miwa, 1979; Maruyama et al, 1987; Nakajima and Nishi, 1989). Some non-Japanese centres have also reported favourably on $D_2$ resections (Smith et al, 1991; Jaehne et al, 1992; Siewert et al, 1993; Sue-Ling et al, 1993; Mendes et al, 1994). However, the benefit of $D_2$ over conventional $D_1$ resections (where only the perigastric nodes within 3.0 cm of the primary are removed) had not been tested prospectively until the launch of the Medical Research Council (MRC) Gastric Cancer Surgical Trial (ST01) in 1986. This was a randomized comparison of $D_1$ versus $D_2$ resections for potentially curable advanced gastric cancer. At the time the study was formulated, the Japanese rules dictated that pancreatico-splenectomy was an integral part of $D_2$ resection for all tumours except antral cancers. For this reason en-bloc removal of these two organs with the stomach was specified by the MRC ST01 trial protocol for middle and upper third tumours in the $D_2$ arm. In this paper, we report on the long-term outcome of these two surgical treatment arms. Preliminary results of ST01 (Cuschieri et al, 1996), and a similar Dutch trial (Bonenkamp et al, 1995), have shown that splenectomy and distal hemi-pancreatectomy are attended by a significant increase in post-operative morbidity and mortality. The influence of removal of these organs on long-term survival is addressed in this analysis. This is important as distal hemi-pancreatectomy is no longer considered an integral part of $D_2$ resections by Japanese surgeons, and some Western centres are practising spleen- and pancreas-preserving $D_2$ resections with apparent good results (Sue-Ling et al, 1993; Griffith, 1995), despite the reported splenic hilar lymph nodes involvement in 15–27% of gastric cancers (Fass and Schumpelick, 1989; Mendes et al, 1994; Mendes et al, 1995; Tsuburaya et al, 1995).

*Received 14 July 1998*
*Revised 20 October 1998*
*Accepted 5 November 1998*

*Correspondence to:* A Cuschieri

### PATIENTS AND METHODS

The organization and preliminary results of the MRC ST01 trial are summarized briefly since they have been reported previously (Cuschieri et al, 1996). Patients enrolled in MRC ST01 were to have histologically proven, and potentially curable, gastric carcinoma. Patients were excluded if they were young (< 20 years), had undergone gastric surgery, harboured a co-existing cancer or had serious co-morbid cardiorespiratory disease that would preclude a safe $D_2$ resection. All patients underwent staging laparotomy to define potentially curative disease. Eligible cases were those that fell within the UICC TNM cancer stages I–III (Sobin and Wittekind, 1997). Tumour stage was determined by pathology of the resected specimens. The patients were randomized centrally (over the telephone), within the same operating session, to either $D_1$ or $D_2$ gastrectomy. In total, 400 eligible patients were randomized.

The operative details of the two arms were defined in terms of the extent of gastric resection, the macroscopic tumour-free margins and the level of lymphadenectomy ($N_1$ or $N_2$). $D_1$ resections entailed removal of the lymph nodes within 3.0 cm of the tumour (considered $N_1$ in TNM system) en bloc with the greater omentum and stomach. $D_2$ resections necessitated the additional removal of the omental bursa, the hepatoduodenal and retroduodenal nodes (antral lesions) and the splenic artery/splenic hilar nodes and retropancreatic nodes by distal hemipancreatico-splenectomy for middle and upper third lesions. In both arms, a distal gastrectomy up to and including the duodenal bulb with a minimum of 2.5 cm proximal tumour-free margin was performed for antral neoplasms, whereas total gastrectomy was undertaken for middle and proximal tumours.

$D_2$ resections were followed by a significantly higher morbidity and mortality than $D_1$ resections; this was attributable on subset analysis to the pancreatico-splenectomy that was largely confined to the $D_2$ arm (Cuschieri et al, 1996). Patients were followed up at regular intervals. Complete follow-up was available to death or 3 years in 96% of patients, and the median follow-up time was 6.5 years. Patients were followed up through the participating surgeon, their General Practitioner (GP) or via the Office for National Statistics.

### Statistical methods

Eligible patients were randomized centrally by use of random permuted blocks, and with stratification for centre, nodal status and tumour location (antral, middle, proximal, total, mixed). Sample size calculations were based on a pre-study survey of 26 gastric surgeons, which indicated that the baseline 5-year survival rate of $D_1$ surgery was expected to be 20%, and improvement in survival to 34% (14% change) with $D_2$ resection would be a realistic expectation. Thus 400 patients (200 in each arm) were to be randomized, providing 90% power to detect such a difference with $P < 0.05$.

The analysis of the trial has been performed on an intention-to-treat basis. The statistical analysis was conducted using the SPSS software system. Univariate survival analyses were performed using the Kaplan–Meier method, and treatment comparisons were made with the log-rank test. Cox's proportional hazards technique was used to fit the multivariate survival model. Significant prognostic factors were chosen using a forward stepwise method.

### RESULTS

The trial profile is shown in Figure 1. In total, 737 patients with histologically proven gastric adenocarcinoma were registered from 32 surgeons in 7 years. Of these, 337 patients were found to be ineligible at staging laparotomy, which confirmed disease at a more advanced stage than that specified in the protocol. Thus 400 eligible patients were randomized, and all were available for analysis,

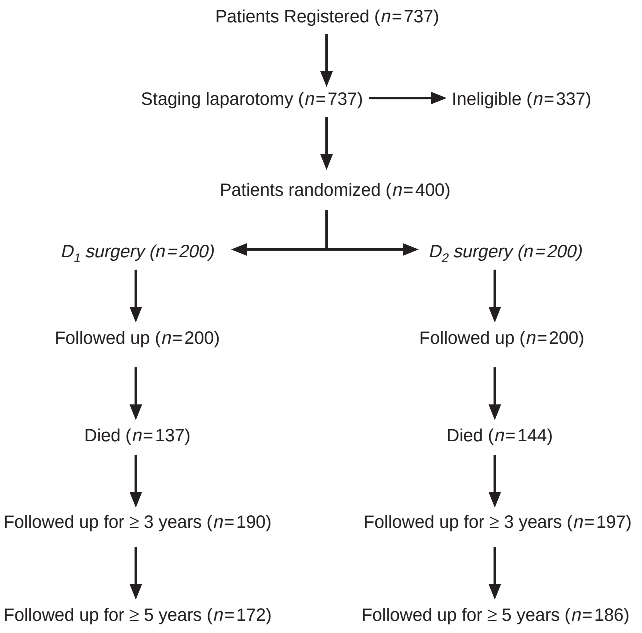

**Figure 1** Trial profile of MRC ST01

although stage could not be ascertained for 25 patients due to missing pathology data. Their characteristics are shown in Table 1.

### Overall survival

The overall 5-year survival rate for both arms is 34% (95% confidence interval (CI) 29–39%).

### Survival by allocated treatment

Survival 'on an intention-to-treat basis' in the two randomized arms of the trial is shown in Figure 2. $D_2$ resection offers no significant survival benefit over $D_1$ surgery (log-rank statistic = 0.63 on 1 degree of freedom (df), $P = 0.43$; hazard ratio (HR) = 1.10, 95% CI 0.87–1.39). The 5-year survival rates are 35% for $D_1$ resection and 33% for $D_2$ resection. Hence the absolute difference in 5-year survival is –2%, and the 95% confidence interval (–12% to 8%) excludes a 5-year survival benefit of more than 8% to $D_2$ resection. The higher post-operative mortality in the $D_2$ arm can be seen by the early dip in the survival curve. It had been thought that improved long-term survival in the $D_2$ arm would compensate for the higher early mortality. However, this does not appear to be the case and the curves have not crossed after 7 years. Survival has also been examined with death from gastric cancer as the event (Figure 3A), post-operative deaths have been censored. Again, there is no benefit to $D_2$ surgery (log-rank statistic = 0.12 on 1 df, $P = 0.72$; HR = 1.05, 95% CI 0.79–1.39). The lack of the early dip in the $D_2$ curve is due to censoring of the post-operative deaths. Similarly, we observed no difference in recurrence-free survival (Figure 3B) between the $D_1$ and $D_2$ groups (log-rank statistic = 0.072 on 1 df, $P = 0.79$; HR = 1.03, 95% CI 0.82–1.29).

### Survival by lymphadenectomy

Within the context of this study, extent of lymphadenectomy can be interpreted as representing 'received' treatment. There is evidence

© Cancer Research Campaign 1999

**Table 1** Patient characteristics

| | $D_1$ surgery | | | $D_2$ surgery | | | Total | | |
|---|---|---|---|---|---|---|---|---|---|
| | *n* | (%) | 5-year survival(%) | *n* | (%) | 5-year survival (%) | *n* | (%) | 5-year survival (%) |
| Total | 200 | | 35 | 200 | | 33 | 400 | | 34 |
| Sex | | | | | | | | | |
| Male | 132 | (66) | 29 | 138 | (69) | 28 | 270 | (67) | 29 |
| Female | 68 | (34) | 45 | 62 | (31) | 44 | 130 | (33) | 45 |
| Age | | | | | | | | | |
| < 60 | 45 | (23) | 54 | 54 | (27) | 47 | 99 | (25) | 50 |
| 60–69 | 78 | (39) | 31 | 61 | (30) | 27 | 139 | (35) | 29 |
| 70 + | 77 | (38) | 28 | 85 | (43) | 29 | 162 | (40) | 29 |
| Location | | | | | | | | | |
| C, CM | 65 | (33) | 24 | 57 | (29) | 17 | 122 | (31) | 21 |
| M, MC, MA | 31 | (15) | 45 | 45 | (22) | 36 | 76 | (19) | 40 |
| A, AM | 91 | (46) | 40 | 94 | (47) | 43 | 185 | (46) | 42 |
| CMA | 13 | (6) | 29 | 4 | (2) | 0 | 17 | (4) | 22 |
| Spleen or pancreas removed? | | | | | | | | | |
| Neither removed | 138 | (69) | 35 | 69 | (35) | 46 | 207 | (52) | 39 |
| Spleen removed | 54 | (27) | 39 | 18 | (9) | 33 | 72 | (18) | 38 |
| Both removed | 8 | (4) | 13 | 113 | (56) | 25 | 121 | (30) | 24 |
| Tumour stage | | | | | | | | | |
| $T_1$ | 48 | (25) | 77 | 40 | (21) | 67 | 88 | (22) | 72 |
| $T_2$ | 63 | (32) | 38 | 69 | (35) | 32 | 132 | (34) | 35 |
| $T_3$ | 84 | (43) | 11 | 86 | (44) | 17 | 170 | (44) | 15 |
| *Missing* | *5* | | | *5* | | | *10* | | |
| Nodal status | | | | | | | | | |
| $N_0$ | 69 | (38) | 63 | 78 | (41) | 51 | 147 | (39) | 56 |
| $N_1$ | 76 | (41) | 16 | 61 | (32) | 25 | 137 | (36) | 20 |
| $N_2$ | 39 | (21) | 21 | 53 | (27) | 13 | 92 | (25) | 17 |
| *Missing* | *16* | | | *8* | | | *24* | | |
| Clinical stage | | | | | | | | | |
| I | 67 | (36) | 69 | 63 | (33) | 58 | 130 | (35) | 64 |
| II | 37 | (20) | 22 | 53 | (28) | 31 | 90 | (24) | 28 |
| III | 80 | (44) | 11 | 75 | (39) | 11 | 155 | (41) | 11 |
| *Missing* | *16* | | | *9* | | | *25* | | |

from the Dutch trial for 'non-compliance or contamination' in the extent of lymphadenectomy performed in the two randomized arms (Bunt et al, 1994). This has occurred in the MRC study as indicated in Table 2, which outlines nodal involvement by location and treatment. The percentage of patients in both arms with involvement of the nodal groups (Table 3) sheds some light on the existing controversies. In the first instance it shows the limited gain in terms of radicality of inclusion of distal pancreatectomy in gastric resections for cancer. Secondly, it documents the widespread nodal involvement in diffuse CMA lesions and, thirdly, it cautions against splenic conservation in proximal tumours.

If a radical lymphadenectomy had been done in accordance with the Japanese rules (Kajitani, 1981), all $D_2$ patients should have had resection of the anterior hepatic nodes (group 8a nodes). Local pathology data are available in 191 $D_2$ patients of whom 95 had documented harvest of these nodes. Survival analysis for $D_1$ vs $D_2$ (hepatic nodes not resected) vs $D_2$ (hepatic nodes resected) showed no significant difference (log-rank statistic = 0.91 on 2 df, $P = 0.63$).

Evaluation of the number of lymph nodes removed is used by the Japanese as a quality control of the extent of lymphadenectomy. The median number of lymph nodes sampled were 13 in the $D_1$ arm and 17 in the $D_2$ arm. Significantly more nodes were sampled in the $D_2$ arm (large scale normal approximation to the Mann–Whitney $U$-test, statistic = –3.98, $P < 0.001$). According to the Japanese rules, a radical lymphadenectomy corresponding to a $D_2$ resection is defined as extirpation of 26 or more nodes (Kajitani, 1981). Of the 375 patients for whom nodal sampling data was available, 310 (165 in the $D_1$ arm, 145 $D_2$) had < 26 nodes and 65 (19 $D_1$, 46 $D_2$) had 26 or more nodes harvested from the specimen by the local pathologist. The survival of these two cohorts of patients was not significantly different (HR = 1.00, 95% CI 0.73–1.37).

### Effect of splenectomy and pancreatico-splenectomy on survival

Table 4 shows the number of patients for each treatment, and tumour location for the patients who had splenectomy only, pancreatico-splenectomy, or neither of these organs removed. No patients had a distal hemipancreatectomy without a splenectomy. Splenectomy was performed in 54 (27%) patients allocated to $D_1$ surgery, the majority ($n = 37$) because of proximal location of the tumour to the spleen where the surgeon considered splenectomy to

 *© Cancer Research Campaign 1999*

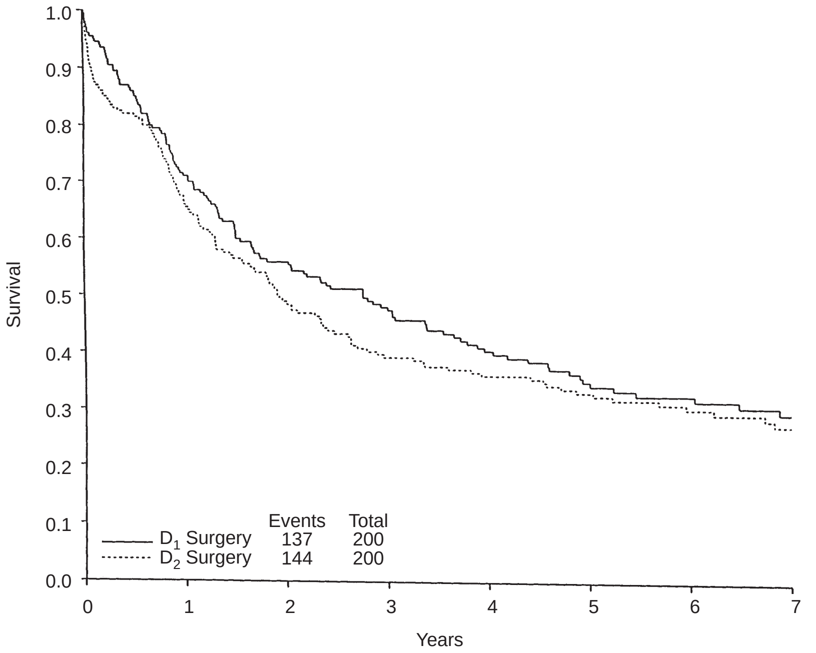

| Patients at risk | Events | | | | | | | | | | | | | | |
|---|---|---|---|---|---|---|---|---|---|---|---|---|---|---|---|
| $D_1$ Surgery | 200 | (58) | 142 | (30) | 108 | (15) | 87 | (13) | 66 | (8) | 48 | (3) | 35 | (3) | 27 |
| $D_2$ Surgery | 200 | (68) | 132 | (34) | 97 | (19) | 76 | (6) | 65 | (5) | 54 | (4) | 36 | (3) | 26 |

**Figure 2** Survival by treatment be necessary. However, seven patients with A, AM tumours also had splenectomy (two for iatrogenic laceration, no reason documented for the others). Four patients in the $D_1$ arm with C, CM lesions had pancreatico-splenectomy. In all instances, this was because of adherence of tumour to the pancreas. In the $D_2$ arm, the patterns of splenic and pancreatic resection reflect the specifications of the protocol, except that 24 patients with A, AM lesions had pancreatico-splenectomy.

Figure 4 shows the survival split for three groups: those with pancreatico-splenectomy (predominantly $D_2$), those with removal of spleen but with preservation of pancreas and those with neither organ removed. There is a significant survival difference between the three groups (log-rank statistic = 9.12 on 2 df, $P = 0.0104$). The pancreatico-splenectomy group had the poorest survival. This adverse effect of pancreatico-splenectomy may be interpreted in light of the lack of benefit to $D_2$ surgery shown by Figures 2 and 3. Since 57% of the $D_2$ arm had pancreatico-splenectomy compared to 4% in the $D_1$ arm, survival of $D_2$ patients who did not have the pancreas removed ought to be better than the corresponding $D_1$ patients to pull the overall curves together. This assumption appears to be strengthened by the survival curve by treatment and splenectomy with or without pancreatectomy (Figure 5). However, the inference that $D_2$ surgery is superior to $D_1$ in this group of patients has to be made cautiously because of the confounding influence of other variables.

### Multivariate analysis

This was undertaken to establish whether splenectomy and pancreatico-splenectomy have an important effect on survival in the presence of other prognostic factors. For example, patients who received $D_2$ surgery without spleen or pancreas removal had better survival than the corresponding $D_1$ group (Figure 5). However, virtually all of these $D_2$ patients had antral tumours, so it could be argued that tumour location is the factor that affects their survival.

The prognostic variables fitted into the model were age, sex, treatment, location of tumour, tumour stage, nodal status, clinical stage, and level of resection of spleen and pancreas. Using a forward stepwise selection procedure, clinical stage, age, sex, and level of resection of spleen and pancreas were found to have a significant influence on survival. The hazard ratios and 95% confidence intervals in the final model are shown in Table 5. Older patients, males and stage II or III patients all experience poor survival. Patients who underwent pancreatico-splenectomy have significantly worse survival than those who had neither organ resected, but the hazard ratios for patients who had spleen alone resected, over those who had neither, falls just short of significance at the 5% level.

It is worth noting that treatment is not an important factor in this model. If it is added to the model, it has a hazard ratio of 0.93 (95% CI 0.68–1.26). However, in the protocol, pancreatico-splenectomy is specified for the majority of $D_2$, but not $D_1$, patients. This analysis thus shows the effect not of allocated treatment but of an ‘idealized’ comparison of $D_1$ with $D_2$, with no imbalance in the proportion of patients undergoing pancreatico-splenectomy.

There is a possibility that nodal status was unbalanced in this trial by the greater nodal sampling in the $D_2$ arm. This would also affect clinical stage. To examine the possible effect of unbalanced nodal status, clinical stage, the multivariate analysis was run without nodal or clinical stage. The resulting model was similar, with tumour stage having the most significant effect instead of clinical

© Cancer Research Campaign 1999

**A**

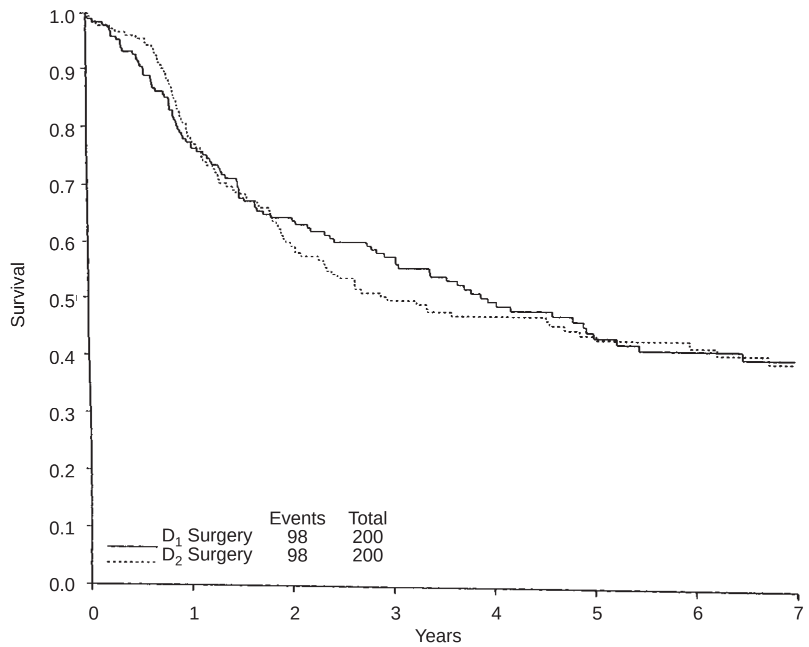

| Patients at risk | Events | | | | | | | | | | | | | |
|---|---|---|---|---|---|---|---|---|---|---|---|---|---|---|
| $D_1$ Surgery | 200 | (42) | 142 | (23) | 108 | (11) | 87 | (11) | 66 | (6) | 48 | (3) | 35 | (1) | 27 |
| $D_2$ Surgery | 200 | (36) | 132 | (31) | 97 | (16) | 76 | (4) | 65 | (4) | 54 | (2) | 36 | (2) | 26 |

**B**

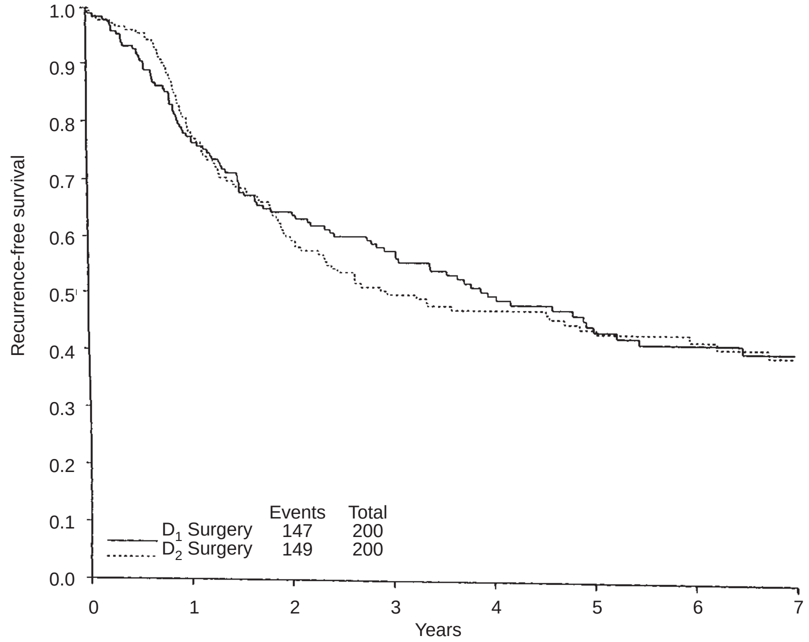

| Patients at risk | Events | | | | | | | | | | | | | |
|---|---|---|---|---|---|---|---|---|---|---|---|---|---|---|
| $D_1$ Surgery | 200 | (77) | 123 | (33) | 86 | (13) | 67 | (9) | 51 | (5) | 37 | (1) | 28 | (4) | 20 |
| $D_2$ Surgery | 200 | (90) | 110 | (26) | 83 | (15) | 66 | (4) | 58 | (5) | 47 | (5) | 29 | (2) | 20 |

**Figure 3** (**A**) Survival by treatment with death from gastric cancer as the event. (**B**) Recurrence-free survival stage. If allocated treatment is added into this model, its hazard ratio is 0.88 (95% CI 0.65–1.20). This is similar to the model with clinical stage, implying that interpretation of any potential treatment effect is not greatly affected by inclusion of clinical stage.

It has been suggested that location of the tumour might have an important effect on survival since location, and level of spleen and pancreas resection are strongly linked. For this reason, the consequences of adding tumour location into the final model was

*© Cancer Research Campaign 1999*

**Table 2** Examination (by local pathologist) and involvement of nodes by location of primary and treatment

| | $D_1$ surgery | | | | $D_2$ surgery | | | |
|---|---|---|---|---|---|---|---|---|
| | C, CM | M, MC, MA | A, AM | CMA | C, CM | M, MC, MA | A, AM[a] | CMA |
| Total number of patients | 64 | 31 | 91 | 13 | 57 | 45 | 94 | 4 |
| Cardiac nodes | | | | | | | | |
| Examined | 39 | 17 | 14 | 7 | 33 | 17 | 15[a] | 2 |
| Involved | 16 | 3 | 2 | 4 | 22 | 2 | 4 | 1 |
| Greater and lesser curve nodes | | | | | | | | |
| Examined | 56 | 28 | 83 | 9 | 52 | 38 | 84 | 4 |
| Involved | 31 | 10 | 39 | 7 | 29 | 16 | 43 | 3 |
| Supra and infra pyloric nodes | | | | | | | | |
| Examined | 32 | 19 | 59 | 8 | 27 | 21 | 61 | 3 |
| Involved | 4 | 4 | 32 | 4 | 5 | 6 | 25 | 0 |
| Left gastric nodes | | | | | | | | |
| Examined | 33 | 15 | 34 | 7 | 41 | 21 | 62 | 3 |
| Involved | 17 | 3 | 11 | 4 | 23 | 4 | 16 | 1 |
| Splenic nodes | | | | | | | | |
| Examined | 16 | 7 | 6 | 4 | 32 | 20 | 19 | 2 |
| Involved | 5 | 1 | 0 | 3 | 7 | 3 | 0 | 0 |
| Hepatic nodes | | | | | | | | |
| Examined | 1 | 3 | 9 | 0 | 28 | 22 | 42 | 3 |
| Involved | 0 | 1 | 3 | 0 | 4 | 3 | 4 | 1 |
| Coeliac nodes | | | | | | | | |
| Examined | 10 | 1 | 9 | 1 | 33 | 19 | 42 | 3 |
| Involved | 4 | 0 | 2 | 0 | 11 | 4 | 8 | 1 |
| Hepato-duodenal nodes | | | | | | | | |
| Examined | 4 | 0 | 7 | 1 | 12 | 16 | 29 | 1 |
| Involved | 0 | 0 | 5 | 1 | 1 | 1 | 3 | 0 |
| Retropancreatic nodes | | | | | | | | |
| Examined | 4 | 0 | 2 | 0 | 17 | 19 | 15 | 3 |
| Involved | 0 | 0 | 1 | 0 | 2 | 4 | 2 | 0 |
| Distant nodes[b] | | | | | | | | |
| Examined | 18 | 3 | 21 | 5 | 14 | 14 | 20 | 1 |
| Involved | 3 | 0 | 1 | 1 | 1 | 1 | 2 | 0 |

$D_1$ = 199 gastric adenoca + 1 lymphoma. [a]Nodes removed in patients with large tumours involving the antrum and middle third. [b]Nodes retrieved from resected specimen > N2 with respect to location of primary.

**Table 3** Percentage of patients in both $D_1$ and $D_2$ arms with nodal involvement by location of tumour

| | C, CM | CMA | M, MC, MA | A, AM[b] |
|---|---|---|---|---|
| Total number of patients | 122 | 17 | 76 | 185 |
| Cardiac nodes | 53[a] (38/72) | 56[a] (5/9) | 15 (5/34) | 20 (6/29)[b] |
| Greater & lesser curve nodes | 56[a] (60/108) | 77[a] (10/13) | 39[a] (26/66) | 49[a] (82/167) |
| Supra & infra pyloric nodes | 15 (9/59) | 36[a] (4/11) | 25[a] (10/40) | 48[a] (57/120) |
| Left gastric nodes | 54[a] (40/74) | 50[a] (5/10) | 19 (7/36) | 28[a] (27/96) |
| Splenic nodes | 25[a] (12/48) | 50[a] (3/6) | 15 (4/27) | – |
| Hepatic nodes | 14 (4/29) | 33[a] (1/3) | 16 (4/25) | 14 (7/51) |
| Coeliac nodes | 35[a] (15/43) | 25[a] (1/4) | 20 (1/4) | 20 (10/51) |
| Hepato-duodenal nodes | 6 (1/16) | 50[a] (1/2) | 6 (1/16) | 22 (8/36) |
| Retro-pancreatic nodes | 10 (2/21) | – | 21 (4/19) | 18 (3/17) |
| Distant nodes[c] | 13 (4/32) | 17 (1/6) | 6 (1/17) | 7 (3/41) |

[a]≥ 1:4 patients with nodal group involvement. [b]Cardiac nodes removed in patients with large tumours involving the antrum and middle third. [c]Nodes retrieved from resected specimen > N2 with respect to location of primary.

*© Cancer Research Campaign 1999*

**Table 4** Spleen and pancreas removal by location and treatment

| Treatment arm | Tumour location | Spleen/pancreas removed? Neither removed Count (%) | Splenectomy only Count (%) | Pancreatico-splenectomy Count (%) |
|---|---|---|---|---|
| $D_1$ surgery | | | | |
| | C, CM | 33 (51) | 28 (43) | 4 (6) |
| | M, MC, MA | 19 (61) | 10 (32) | 2 (7) |
| | A, AM | 83 (91) | 7 (8) | 1 (1) |
| | CMA | 3 (23) | 9 (69) | 1 (8) |
| | Total | 138 (69) | 54 (27) | 8 (4) |
| $D_2$ surgery | | | | |
| | C, CM | 1 (2) | 7 (12) | 49 (86) |
| | M, MC, MA | 2 (4) | 7 (16) | 36 (80) |
| | A, AM | 66 (70) | 4 (4) | 24 (26) |
| | CMA | 0 (0) | 0 (0) | 4 (100) |
| | Total | 69 (34) | 18 (9) | 113 (57) |

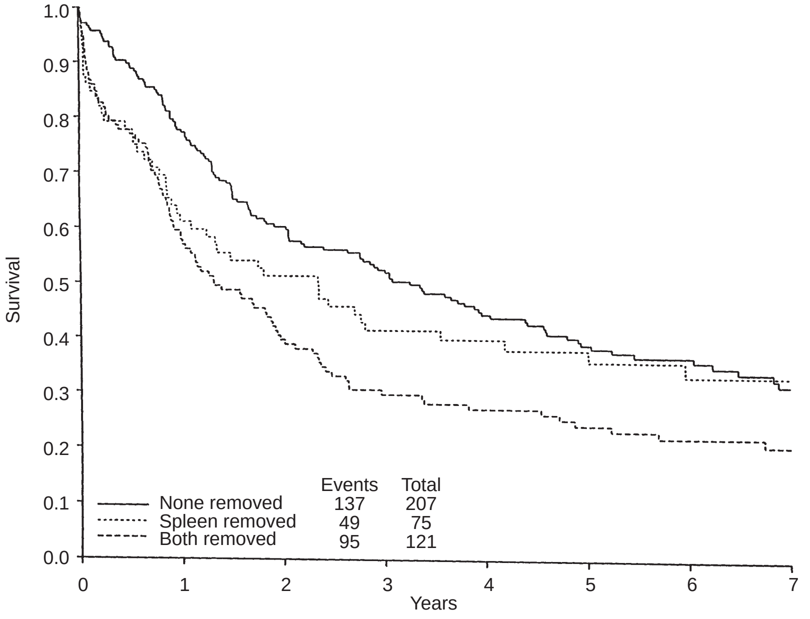

| Patients at risk | Events | | | | | | | | | | | | |
|---|---|---|---|---|---|---|---|---|---|---|---|---|---|
| None removed | 207 | (47) | 160 | (35) | 120 | (15) | 99 | (15) | 78 | (9) | 59 | (3) | 40 | (5) | 27 |
| Spleen removed | 72 | (28) | 44 | (7) | 37 | (7) | 28 | (1) | 23 | (1) | 18 | (2) | 13 | (0) | 12 |
| Both removed | 121 | (51) | 70 | (22) | 48 | (12) | 36 | (3) | 30 | (3) | 25 | (2) | 18 | (1) | 14 |

**Figure 4** Survival by spleen and pancreas removal investigated. Adding tumour location does not greatly alter the hazard ratio for splenectomy alone (HR = 1.23) and for pancreatico-splenectomy (HR = 1.47), and the estimate of treatment effect (HR = 0.96) does not change significantly.

### DISCUSSION

This trial has shown that there is no difference in long-term survival between $D_1$ and $D_2$ surgery equivalent to the Japanese $D_2$ resection as defined by JRSGC involving pancreatico-splenectomy for middle and proximal third tumours. It could be argued that the $D_1$ resection (based on TNM system, i.e. removal of lymph nodes within a 3.0 cm radius of the primary) in this trial did not conform with the strict definition of the JRSGC (removal of $N_1$ lymph nodes in accordance with location of primary). In essence, however, these are equivalent in terms of the nodal harvest, although some argue that the TNM-based $D_1$ resection removes more nodes than the Japanese equivalent. There are no comparative trials to confirm this view. The other problem inherent to all surgical trials has been ‘contamination and non-compliance’. In the MRC study we relied on individual responsibility of the participating surgeons who were shown videos of the procedures and had agreed to undertake the two surgical options, highlighted in a booklet designed for the study. Quality control could only be

 *© Cancer Research Campaign 1999*

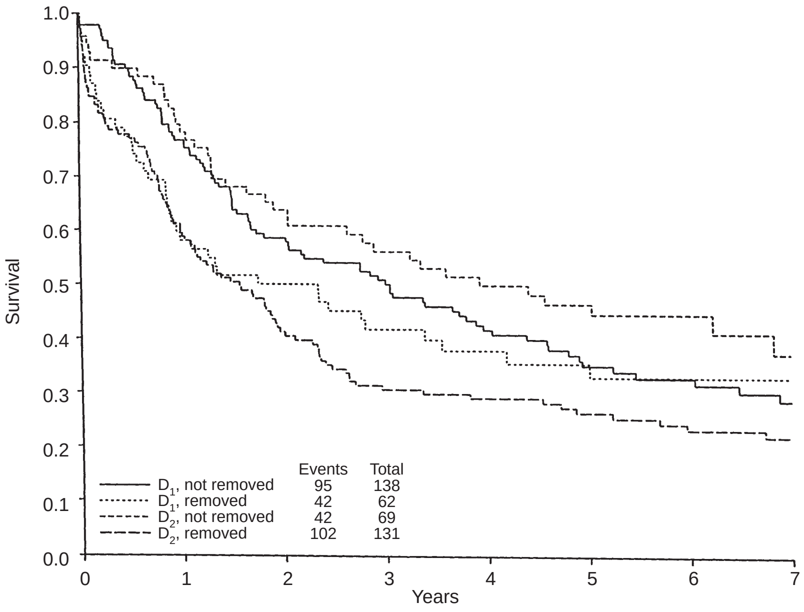

| Patients at risk | | Events | | | | | | | | | | | | | |
|---|---|---|---|---|---|---|---|---|---|---|---|---|---|---|---|
| $D_1$, not removed | 138 | (32) | 106 | (25) | 77 | (10) | 63 | (11) | 48 | (7) | 34 | (2) | 26 | (3) | 18 |
| $D_1$, removed | 62 | (26) | 36 | (5) | 31 | (5) | 24 | (2) | 18 | (1) | 14 | (1) | 9 | (0) | 9 |
| $D_2$, not removed | 69 | (15) | 54 | (10) | 43 | (5) | 36 | (4) | 30 | (2) | 25 | (1) | 14 | (2) | 9 |
| $D_2$, removed | 131 | (53) | 78 | (24) | 54 | (14) | 40 | (2) | 35 | (3) | 29 | (3) | 22 | (1) | 17 |

**Figure 5** Survival by treatment with spleen and distal pancreas removed or not removed

**Table 5** Hazards ratios and 95% CIs for the fitted multivariate model

| Variable | Hazard ratio | 95% CI | *P*-value |
|---|---|---|---|
| Clinical stage | | | |
| I | 1.0 | | |
| II | 2.19 | 1.54–3.12 | < 0.0001 |
| III | 3.87 | 2.83–5.28 | < 0.0001 |
| Age | 1.03 | 1.01–1.04 | 0.0001 |
| Sex | | | |
| Male | 1.0 | | |
| Female | 0.62 | 0.48–0.81 | 0.0005 |
| Spleen/pancreas resection | | | |
| Neither resected | 1.0 | | |
| Splenectomy only | 1.36 | 0.97–1.90 | 0.0716 |
| Pancreatico-splenectomy | 1.53 | 1.17–2.01 | 0.0020 |

assessed by the operative data forms (site of tumour, extent of gastric resection, resection margins, lymph node harvest) and the pathological examination of the resected specimens with respect to location of the tumour. Supervision of the surgeons in the operating room was not possible. Despite this obvious limitation, the problem of contamination and non-compliance does not appear to be greater than that encountered in the Dutch study (Bunt et al, 1994) where an experienced Japanese surgeon proctored the participating surgeons for some time.

The mortality reported for the $D_1$ and $D_2$ arms of the MRC ST01 study is virtually identical to that of the equivalent Dutch trial, but undoubtedly higher than that reported by the Japanese (Mine et al, 1970; Miwa, 1979; Maruyama et al, 1987; Nakajima and Nishi, 1989) and some Western centres (Smith et al, 1991; Jaehne et al, 1992; Siewert et al, 1993; Sue-Ling et al, 1993; Mendes et al, 1994). Whilst factors such as experience born of sustained case-load, surgical skill, quality of post-operative care and case selection are important, it is not possible to make valid comparisons on mortality between published series without data on pre-operative risk stratification of the patients. Subset analysis of the surgeons' results in the ST01 study showed no effect of caseload (number of patients entered) on post-operative mortality.

The other main conclusion reached is that pancreatico-splenectomy should not form a routine part of $D_2$ resections. Pancreatico-splenectomy appears to disadvantage these patients, both in terms of increased post-operative morbidity and mortality and, probably, by reducing long-term survival. Undoubtedly, the high proportion of pancreatico-splenectomies in the $D_2$ arm must have adversely affected the overall survival rate of $D_2$ patients. Hence $D_2$ surgery without pancreatico-splenectomy may carry better survival rates than $D_1$ resection, but this inference must be tested by a further randomized study. It is difficult to untangle the adverse effects of splenectomy from those of pancreatectomy on the survival of patients in this trial but the multivariate analysis suggests that pancreatic resection has the stronger effect. The recommendation is, therefore, that pancreatic resection should only be performed in $D_2$ resections if there is direct extension of disease to the pancreas from posteriorly situated tumours. Preservation of the pancreas is now being recommended and practised by Japanese surgeons in $D_2$ resections for gastric cancer (Otsuji et al, 1997).

It is difficult to reach any definite conclusions on the influence of the extent of lymphadenectomy on long-term survival. The comparison in the present trial between radical lymphadenectomy, as defined by the Japanese rules, and those with nodal harvest of

© Cancer Research Campaign 1999

25 or fewer regional nodes suggests no difference but it must be stressed that this analysis was conducted on non-randomized data. In the German prospective, but non-randomized, study where the surgeons were allowed to perform their preferred resection, radical lymphadenectomy (26 or more nodes in the specimen) significantly improved survival only in stage II and stage IIIa disease (Siewert et al, 1993). This effect was, however, restricted to patients with $pN_0$ and $pN_1$. There is strong evidence that pancreatico-splenectomy and splenectomy alone (and not radical lymphadenectomy) are responsible for the increased morbidity and mortality in the $D_2$ arm of the MRC ST01 study (Cuschieri et al, 1996) and this observation is in agreement with the findings of the Dutch trial (Bonenkamp et al, 1995). In the MRC ST01 trial, the best long-term survival was obtained in the subgroup of patients who underwent $D_2$ resection without pancreatico-splenectomy. Given the evidence that pancreatectomy is detrimental and should be avoided unless necessary because of local involvement of the pancreas during $D_2$ resections, the question then remains of whether the spleen should be preserved or removed during $D_2$ resections for proximal gastric cancer. Two centres in the UK have reported impressive results with low post-operative morbidity and mortality and improved survival with spleen preserving $D_2$ resections (Sue-Ling et al, 1993; Griffith, 1995). The argument against splenic preservation is the incidence of lymph node metastases along the distal splenic artery and splenic hilium (Japanese groups 10, 11) in patients with proximal tumours. Reported estimates for deposits in these nodes vary from 15 to 27% (Fass and Schumpelick, 1989; Mendes et al, 1994, 1995; Tsuburaya et al, 1995). In the present study 25% of patients with C and CM tumours had involved splenic nodes. Some, possibly all, of the splenic artery nodes can be removed with preservation of the pancreas and spleen although this requires a high level of technical skill, but the splenic hilar nodes cannot be removed safely without a splenectomy. This is the remaining issue to be resolved in surgery for proximal gastric cancer (excluding CMA lesions) – radical lymphadenectomy with spleen preservation or splenectomy. Aside from post-operative morbidity, the risks of fulminating post-splenectomy infections are well-documented, and the possibility of increased growth of micrometastases in splenectomized patients cannot be dismissed.

### ACKNOWLEDGEMENT

The authors would like to thank Sally Stenning (MRC Cancer Division, Clinical Trials Unit) for her helpful comments and input during the preparation of the manuscript.

### REFERENCES

Bonenkamp JJ, Songun I, Herman J,Sasako M, Welvaart K, Plukker JT, van Elk P, Obertop H, Gouma DJ, Taat CW, van Lanschot J, Meyer S, de Graaf PW, von Meyenfeldt MF, Tilanus H and van de Velde CJH (1995) Randomised comparison of morbidity after D1 and D2 dissection for gastric cancer in 996 Dutch patients. *Lancet* **345**: 745–748

Bunt AMG, Bonenkamp HJ, Herman J, van de Velde CJ, Arends JW, Fleuren G and Bruijn JA (1994) Factors influencing noncompliance and contamination in a randomised trial of 'Western' (r1) versus 'Japanese' (r2) type surgery in gastric cancer. *Cancer* **73**: 1544–1551

Cuschieri A, Fayers P, Fielding J, Craven J, Bancewicz J, Joypaul V and Cook P (1996). Post-operative morbidity and mortality after D1 and D2 resections for gastric cancer: preliminary results of the MRC randomised controlled surgical trial. *Lancet* **347**: 995–999

Fass J and Schumpelick V (1989) Principles of radical surgery in gastric carcinoma. *Hepatogastroenterology* **36**: 13–17

Griffith JP (1995). Preservation of the spleen improves survival after radical surgery for gastric cancer. *Gut* **36**: 684–690

Jaehne J, Meyer HJ, Maschek H, Geerlings H, Bruns E and Pichlmayr R (1992) Lymphadenectomy in gastric carcinoma. *Arch Surg* **127**: 290–294

Kajitani T (1981) Japanese Research Society for Gastric Cancer. The general rules for the gastric cancer study in surgery and pathology. *Jpn J Surg* **11**: 127–139

Maruyama K, Okabayashi K and Kinoshita T (1987) Progress in gastric cancer surgery and its limits of radicality. *World J Surg* **11**: 418–426

Mendes de Almeida JC, Bettencourt A, Santos Costa C and Mendes de Almeida JM (1994) Curative surgery for gastric cancer: study of 166 consecutive patients. *World J Surg* **18**: 889–895

Mendes de Almeida JC, Bettencourt A, Santos Costa C and Mendes de Almedida JM (1995) Impact of distal pancreatectomy and splenectomy in D2 dissection for gastric cancer. In *First International Gastric Cancer Congress 1995*, vol 2, pp. 1165–1169. Monduzzi Editore SpA: Bologna

Mine M, Majima S, Harada M and Etani S (1970) End results of gastrectomy for cancer: effect of extensive lymph node dissection. *Surgery* **68**: 753–758

Miwa K (1979) Cancer of the stomach in Japan. *Gann Monogr Cancer Res* **22**: 61–75

Nakajima T and Nishi M (1989) Surgery and adjuvant chemotherapy for gastric cancer. *Hepatogastroenterology* **36**: 79–85

Otsuji E, Yamaguchi T, Sawai K, Okamato T and Takahashi T (1997). End results of simultaneous pancreatectomy, splenectomy and total gastrectomy for patients with gastric carcinoma. *Br J Cancer* **75**: 1219–1223

Siewert JR, Bottcher K, Roder JD, Busch R, Hermanek P and Meyer HJ (1993) Prognostic relevance of systematic node dissection in gastric carcinoma. *Br J Surg* **80**: 1015–1018

Smith JW, Shiu MH, Kelsey L and Brennan MF (1991) Morbidity of radical lymphadenectomy in the curative resection of gastric carcinoma. *Arch Surg* **126**: 1469–1473

Sobin LH and Wittekind Ch (eds) (1997) *UICC TNM Classification of Malignant Tumours*, 5th Edn. John Wiley and Sons: New York

Sue-Ling HM, Johnston D, Martin IG, Dixon MF, Lansdown MR, McMahon MJ and Axon AT (1993) Gastric cancer: a curable disease in Britain. *Br Med J* **307**: 591–596

Tsuburaya A, Sairebji M, Kobayashi O, Taniguchi T and Motohashi H (1995) Impact of pancreatico-splenectomy on survival and quality of life of patients with gastric cancer. In *First International Gastric Cancer Congress 1995*, vol 2, pp. 1177–1179. Monduzzi Editore SpA: Bologna

### APPENDIX

### Members of the Surgical Cooperative Group

W Allum (UK), J Bancewicz (UK), HD Becker (Germany), A Broughton (UK), FC Campbell (UK), J Clark (UK), J Craven (Jamaica), A Cuschieri (UK), A Cook (UK), I Donovan (UK), N Dorricot (UK), D Ellis (UK), J Fielding (UK), P Finan (UK), D Fossard (UK), A Hall (UK), M Hallisey (UK), T Hennessey (Ireland), D Kumar (UK), J Magnusson (Iceland), M Mughal (UK), G Sagor (UK), O Soreide (Norway), R Stedeford (UK), S Stipa (Italy), C Stoddard (UK), T Taylor (UK).

© Cancer Research Campaign 1999
